# Atorvastatin can delay arterial stiffness progression in hemodialysis patients

**DOI:** 10.1007/s11255-022-03231-3

**Published:** 2022-05-18

**Authors:** Mohamed Mamdouh Elsayed, Elhassan Mohamed Ayman

**Affiliations:** Nephrology and Internal Medicine Department, Faculty of Medicine, Alexandria University, Alkhartoom square, El azareeta, Alexandria, 21131 Egypt

**Keywords:** Atorvastatin, Arterial stiffness, Augmentation index, Hemodialysis, Pulse wave velocity

## Abstract

**Purpose:**

Arterial stiffness is one of the vascular pathologies in hemodialysis (HD) patients with increased cardiovascular mortality and morbidity. Few approaches have been tested to reduce arterial stiffness in patients with chronic kidney disease (CKD). We aimed to assess effects of atorvastatin on arterial stiffness in hemodialysis patients.

**Methods:**

This research is a double-blinded, placebo-controlled, randomized clinical trial which included 50 patients maintained on regular HD. Patients were allocated to receive 10 mg atorvastatin or placebo for 24 weeks. Aortic pulse wave velocity (PWV) as an index of large artery stiffness and augmentation index (AIx) as an index of wave reflections were assessed at baseline and after 6 months in both groups.

**Results:**

In atorvastatin group at study end, there was no significant difference from baseline findings in aortic PWV (7.86 ± 2.5 vs 7.88 ± 2.6 m/sec; *p* = 0.136), AIx (26.04 ± 8.5 vs 26.0 ± 8.6%; *p* = 0.714) and central pulse pressure (PP) (*p* = 1.0). On the other hand, in placebo group after 24 weeks, aortic PWV (7.80 ± 2.16 vs 7.63 ± 2.1 m/sec; *p* < 0.001), AIx (25.88 ± 9.4 vs 25.04 ± 9.4%; *p* < 0.001) increased significantly from baseline measurements but central pulse pressure (PP) (*p* = 0.870) did not. Also, the change (Δ) in aortic PWV and AIx was significantly higher than the change in the atorvastatin group with *p* value of < 0.001 and < 0.001, respectively.

**Conclusions:**

Arterial stiffness parameters remained stable in atorvastatin group but increased significantly in placebo-treated patients suggesting a potential role for atorvastatin to delay arterial stiffness progression in HD patients. Larger randomized clinical trials are needed to confirm these findings.

**Clinical Trials registration:**

ClinicalTrials.gov NCT04472637.

## Introduction

Arterial stiffness (AS) is associated with increased cardiovascular mortality and morbidity [1]. Compared to normal population, AS occurs at an accelerated rate in patients with chronic kidney disease (CKD) and end-stage renal disease (ESRD) [2]. Vascular calcifications in ESRD patients aggravate AS [3]. Many factors have been incriminated in the pathogenesis including uremic toxins, premature vascular aging, metabolic, hormonal, and inflammatory factors [2]. AS can be assessed noninvasively with the use of the aortic pulse wave velocity (PWV) as an index of large artery stiffness and augmentation index (AIx) as an index of wave reflections [4].

In CKD population, reducing AS was associated with improved survival [5]. Many drugs have been studied to improve AS with variable degrees of success including antihypertensive medications, anti-inflammatory drugs, endothelin-1 antagonist, antioxidants, immunosuppressive drugs, and statins [6–11].

Beneficial effects of statins in reducing cardiovascular events in general population have been well documented in many guidelines [12]. Potential mechanisms include improvement in lipid profile, endothelial function, vascular inflammation, and AS [13]. Many authors found that statins reduce AS in patients with hypertension, hypercholesterolemia, and diabetes mellitus [14, 15]. There is paucity of data regarding effects of statins on arterial stiffness in CKD population. However, some have found encouraging results with statin use [11]. To our knowledge, we are the first study to assess effects of atorvastatin on arterial stiffness exclusively in non-diabetic hemodialysis (HD) patients.

## Materials and methods

### Participants

This research is a double-blinded, placebo-controlled, randomized clinical trial which enrolled 50 patients maintained on regular HD in Alexandria main university hospital and Al Mowasa University Hospital for more than 3 months. They perform thrice weekly, 4 h HD sessions to achieve a target Kt/V of at least 1.4. Patients were randomly assigned using block randomization method to receive 10 mg atorvastatin or placebo for 24 weeks. Participants, health care providers, as well as the outcome assessor were unaware about the type of treatment each patient received. Allocation concealment was ensured using sealed closed envelop randomization technique and every patient was given an identification code. Patients with diabetes mellitus, severe valvular heart disease, irregular heart rhythm, history of aortic surgery/prosthetic aorta, acute liver disease, history of myocardial infraction in the previous 6 months, pregnancy, and those receiving lipid lowering drugs were excluded from the study. The trial was registered on Clinicaltrials.gov (NCT04472637).

### Methods and study outcomes

All patients were subjected to full history taking including cause of ESRD, duration of HD, and full clinical examination. Laboratory investigations included serum triglycerides, total cholesterol, high-density lipoprotein (HDL), and low-density lipoprotein (LDL).

Arterial stiffness indices and central blood pressure (BP) were assessed using Mobil-O-Graph NG device (I.E.M. GmbH, Stolberg, Germany) [16]. It is an oscillometric ambulatory BP monitoring device, whose brachial BP-detection unit was validated according to the standard protocols [17]. Assessment was done early in the morning and 1 h before the midweek HD session. Smoking and caffeine were not allowed for at least 2 h before examination. A suitable cuff was placed in the non-fistula arm after 10 min rest in supine position. The cuff is linked to a recorder device and all signals obtained were transmitted to a computer for analysis and interpretation. Then, through an analyzing software (ARCSolver) program, brachial BP measurements were transformed into aortic pulse waveform. Aortic systolic blood pressure (SBP), diastolic blood pressure (DBP), and pulse pressure (PP) were obtained after analysis of these waves. Augmentation index was also measured to assess peripheral artery stiffness. These measurements were recorded at baseline and after 6 months in both groups.

### Statistical analysis

Data were fed to the computer and analyzed using IBM SPSS software package version 20.0. (Armonk, NY: IBM Corp). Categorical data were represented as numbers and percentages. Chi-square test was applied to investigate the association between the categorical variables. For continuous data, they were tested for normality by the Shapiro–Wilk test. Distributed data were expressed as mean and standard deviation. Student’s *t* test was used to compare two groups for normally distributed quantitative variables, while paired *t* test was used to compare between two periods. On the other hand, Mann–Whitney test was used to compare two groups for abnormally distributed quantitative variables. Significance of the obtained results was judged at the 5% level. For purposes of sample size calculation, the outcome variable used was the aortic PWV. An effect size of 1.2 was assumed to be clinically significant. With a pooled standard deviation of approximately 1.5, statistical power calculations were performed by means of two-sample t test using a significance level of 5% and a two sided alternative hypothesis. The calculations resulted the need to recruit 25 patients in each group to reach a statistical power of 80% to detect a difference of 1.2 between the placebo and intervention groups. Power analysis was conducted using the R programming language.

## Results

### Baseline characteristics of patients

**Fig. 1:**
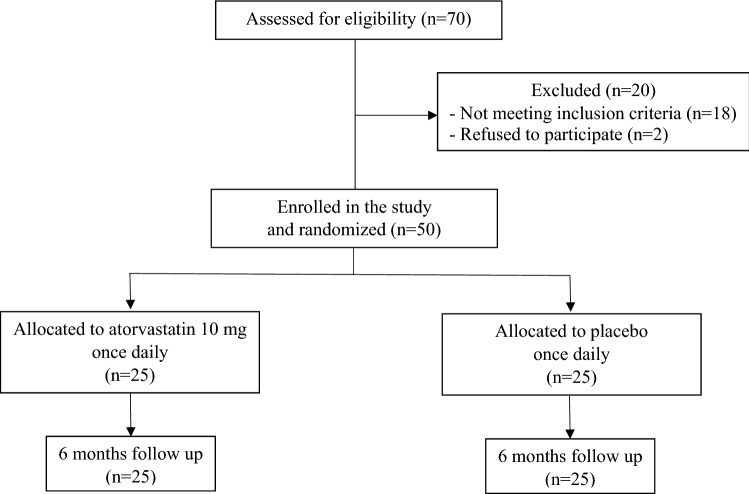
Patient enrollment flow diagram

Seventy HD patients were assessed to participate in the study. Of these, 18 did not meet inclusion criteria and 2 refused to participate. In total, 50 HD patient were enrolled in the study. After randomization, 25 patients received 10 mg atorvastatin and the other 25 patients received a placebo for 24 weeks (Fig. 1). Clinical characteristics of patients are displayed in Table 1. There was no statistically significant difference between both groups regarding age, sex, body mass index (BMI), blood pressure, smoking status, cause of ESRD, duration of HD, antihypertensive medications, and lipid profile.

**Table 1:** Baseline characteristics of the study group

	Atorvastatin group (*n* = 25)	Placebo group (*n* = 25)	*p* Value
Age (years)	47.72 ± 10.26	47.20 ± 10.81	0.862
Sex (no)			
Male	15 (60%)	12 (48%)	0.395
Female	10 (40%)	13 (52%)	
BMI (kg/m^2^)	27.26 ± 5.09	26.84 ± 4.03	0.750
Smokers (no) %	6 (24%)	7 (28%)	0.747
Blood pressure (mm/Hg)			
Systolic	133.6 ± 28.27	130.2 ± 24.52	0.652
Diastolic	80.20 ± 13.42	77.20 ± 16.21	0.479
Duration of HD (years)	7.42 ± 4.75	6.88 ± 6.08	0.403
Cause of ESRD (no)			
Hypertension	8	9	
Glomerulonephritis	3	4	
APCKD, congenital	6	5	
Others		7	
Dialysis modality (no)			
HD	24	24	
HDF	1	1	
Comorbidities (no)			
Hypertension	11	10	
Heart failure	3	2	
Asthma	2	3	
COPD	1	2	
Anti-hypertensive drugs (no)			
Beta blockers	3	3	
CCBs	4	5	
ACEI, ARBS	1	1	
Phosphate binders (no)			
Calcium Based	12	13	
Non-calcium-based	2	2	
Alfacalcidol use (no)	10	11	
Calcimimetics use (no)	2	2	
Total cholesterol (mg/dl)	175.5 ± 27.15	183.3 ± 24.48	0.289
LDL cholesterol (mg/dl)	98.48 ± 19.94	94.60 ± 19.14	0.486
HDL cholesterol (mg/dl)	38.88 ± 8.04	37 ± 7.18	0.387
Serum triglycerides (mg/dl)	143 ± 39.67	138.9 ± 42.30	0.727
Hemoglobin (g/dl)	9.85 ± 1.26	9.7 ± 1.23	0.784
Serum albumin (g/dl)	3.85 ± 0.31	4.02 ± 0.87	0.325
SGPT (u/l)	25.25 ± 5.47	22.64 ± 4.21	0.737
Serum calcium (mg/dl)	8.82 ± 1.13	8.71 ± 1.24	0.656
Serum phosphorus (mg/dl)	5.61 ± 1.42	5.47 ± 1.07	0.563
Serum PTH (pg/ml)	518.64 ± 542.53	509.24 ± 530.43	0.476
Kt/V	1.41 ± 0.31	1.42 ± 0.45	0.786

Data were expressed as mean ± standard deviation (SD), or absolute numbers as appropriate

*ACEI* angiotensin-converting enzyme inhibitors, *ARBS *angiotensin receptor blockers, *APCKD* adult polycystic kidney disease, *BMI* body mass index, *COPD* chronic obstructive lung disease, *HDF* hemodiafiltration, *Kt/V* measuring dialysis adequacy, *PTH* parathyroid hormone, *SGPT* serum glutamic pyruvic transaminase

### Arterial stiffness, wave reflection, and peripheral and central blood pressure parameters

**Fig. 2:**
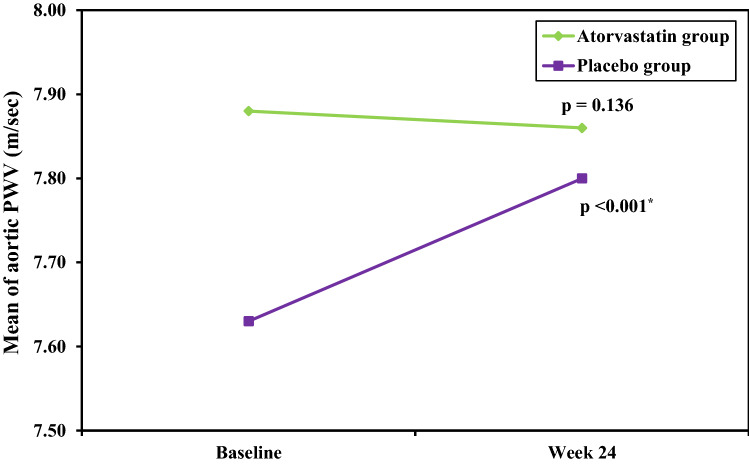
Change in aortic pulse wave velocity (PWV) during study period in each group

**Fig. 3:**
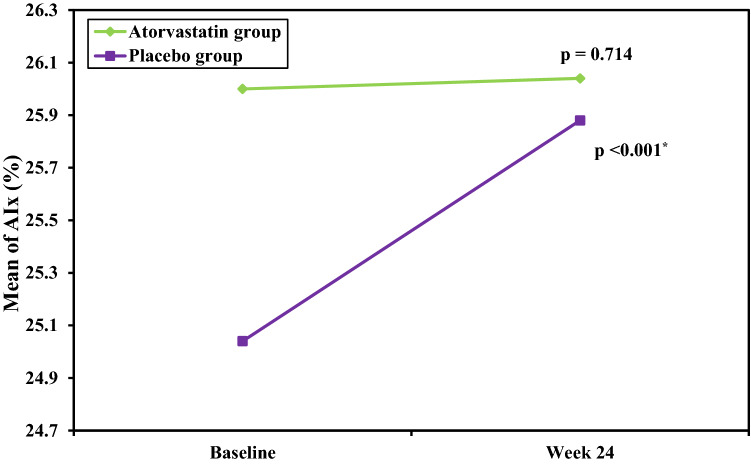
Change in augmentation index (AIx) during study period in each group

At baseline, aortic PWV and AIx values showed no significant difference between both groups. At study end, aortic PWV and AIx remained stable in the atorvastatin group with *p* value of 0.136 and 0.714, respectively, but showed a significant increase in the placebo group with *p* value of < 0.001 and < 0.001, respectively. Also, the change (Δ) in aortic PWV and AIx in the placebo group was significantly higher than the change in the atorvastatin group with *p* value of < 0.001 and < 0.001, respectively (Table 2), (Figs. 2, 3).

**Table 2:** Arterial stiffness and wave reflection parameters at baseline and study end in both groups

	Atorvastatin group (*n* = 25)				Placebo group (*n* = 25)				Comparison between groups		
	Baseline	Week 24	*p*_0_	Change (Δ)	Baseline	Week 24	*p*_0_	Change (Δ)	*p*_*1*_	*p*_2_	*p*_3_
Aortic PWV (m/sec)	7.88 ± 2.60	7.86 ± 2.57	0.136	−0.02 ± 0.08	7.63 ± 2.14	7.80 ± 2.16	< 0.001	0.17 ± 0.05	0.706	0.929	< 0.001
AIx (%)	26.0 ± 8.61	26.04 ± 8.58	0.714	0.04 ± 0.54	25.04 ± 9.48	25.88 ± 9.42	< 0.001	0.84 ± 0.47	0.709	0.950	< 0.001

Data were expressed in Mean ± SD. *p*_0_: p value for comparing between Baseline and Week 24 in each group

*DBP* diastolic blood pressure, *PP* pulse pressure, *SBP* systolic blood pressure

*p*_1_: *p* value for comparing between Atorvastatin group and Placebo group at Baseline

*p*_2_: *p* value for comparing between Atorvastatin group and Placebo group at Week 24

*p*_3_: *p* value for comparing the change (delta) between Atorvastatin group and Placebo group

Table 3 displays peripheral and central blood pressure parameters (SBP, DBP, PP) that were not significantly different between atorvastatin and placebo groups either at baseline or after 24 weeks. At the end of the study, these parameters in each group showed nonsignificant difference from baseline values, Table 3.

**Table 3:** Peripheral and central blood pressure parameters at baseline and study end in both groups

	Atorvastatin group (*n* = 25)			Placebo group (*n* = 25)			Comparison between groups	
	Baseline	Week 24	*p*_0_ value	Baseline	Week 24	*p*_0_ value	*p*_1_	*p*_2_
Brachial SBP (mmHg)	137.0 ± 21.69	138.2 ± 20.36	0.863	135.4 ± 23.18	137.0 ± 20.82	0.212	0.797	0.838
Brachial DBP (mmHg)	81.40 ± 14.11	82.0 ± 13.07	0.859	80.40 ± 14.43	82.0 ± 11.46	0.175	0.805	1.000
Brachial PP	55.64 ± 28.08	56.20 ± 26.47	0.947	55 ± 30.79	55 ± 27.8	1.000	0.939	0.876
Aortic SBP (mmHg)	123.4 ± 20.14	123.2 ± 13.53	0.968	121.2 ± 20.07	122.0 ± 20.05	0.444	0.701	0.805
Aortic DBP (mmHg)	78.20 ± 13.99	78.0 ± 12.50	0.832	76.40 ± 11.68	77.0 ± 12.58	0.417	0.624	0.779
Aortic PP	45.20 ± 21.96	45.20 ± 16.42	1.000	44.80 ± 18.79	45 ± 17.68	0.870	0.945	0.967
Heart rate (beats/min.)	71.52 ± 7.91	71.0 ± 7.44	0.382	73.20 ± 8.69	72.36 ± 9.05	0.174	0.478	0.564

Data were expressed in Mean ± SD. *p*_0_: *p* value for comparing between Baseline and Week 24 in each group

*DBP* diastolic blood pressure, *PP* pulse pressure, *SBP* systolic blood pressure

*p*_1_: *p* value for comparing between Atorvastatin group and Placebo group at Baseline

*p*_2_: *p* value for comparing between Atorvastatin group and Placebo group at Week 24

## Discussion

This study is the first randomized clinical trial to study the impact of low dose atorvastatin on AS in non-diabetic HD patients. Our main finding was that arterial stiffness parameters increased significantly in placebo group but not in atorvastatin treated patients.

AS plays a major role in the pathogenesis of CVD in persons with normal kidney function, but this role is much higher in CKD patients [18]. This hardening exaggerates with CKD progression [2]. The final statement about the effect of atorvastatin on AS is not clear, because the results of the trials are conflicting. Some showed improvement [19, 20], but others revealed no change [21] or even deterioration with statin use [22].

Only few studies have been done in CKD population. Fasset et al. enrolled 37 patients with CKD and found similar results to our study reporting a significant increase in AS in placebo group but not in atorvastatin group after 36 months [11]. They differ from our study in that they included patients with early stages of CKD with a mean serum creatinine of 2 mg/dl. Ichihara et al. in their study which included 22 HD diabetic patients found that fluvastatin use for 6 months significantly reduced PWV in the treatment group [23]. The main drawback of this study was that they assessed PWV in peripheral arteries not in the aorta which is the gold standard.

Pathogenesis of AS in ESRD is multifactorial with vascular calcifications playing a major role higher than in other medical conditions [3]. In advanced CKD, there is imbalance between inhibitors and promotors of vascular calcifications [24]. Another characteristic condition in ESRD is that HD induces a chronic inflammatory state [25]. This chronic inflammation through increased tumor necrosis factor alpha (TNF) levels, and increased reactive oxygen species (ROS) causing endothelial dysfunction with reduced nitic oxide (NO) levels leads to proliferation and phenotypic switch of vascular smooth muscle cells (VSMCs) [26, 27]. There might be potential mechanisms for the beneficial effects we found with atorvastatin use. Atorvastatin has an anti-inflammatory action through reducing ROS [28] and improves endothelial function by increasing NO availability leading to decreased vascular tone [29]. This effect on vascular tone is augmented by antagonizing endothelin-1 mediated vasoconstriction [30].

Regarding atorvastatin safety, liver enzymes (SGOT, SGPT) were withdrawn every 2 months and no elevations occurred. Also, there was no reporting of muscle pain or weakness. Low dose of atorvastatin used (10 mg/day) might explain this safe profile.

The strengths of our study include being first to investigate atorvastatin impact on AS in HD patients with a considerable follow-up period. Assessing AS through central arteries not peripheral ones which is the standard method. A possible drawback of our study might be the follow-up period (6 months), although it is the longest period till now, but longer durations will strengthen the findings. Also, PWV was assessed statically not ambulatory which is more preferred.

## Conclusion

In conclusion, AS parameters remained stable in atorvastatin group but increased significantly in placebo-treated patients. These findings might suggest a potential role for atorvastatin to delay arterial stiffness progression in HD patients. Larger randomized clinical trials for a longer follow-up periods are needed to confirm these findings.
